# Local and systemic effects of cat allergen nasal provocation

**DOI:** 10.1111/cea.12434

**Published:** 2015-02-25

**Authors:** G. W. Scadding, A. Eifan, M. Penagos, A. Dumitru, A. Switzer, O. McMahon, D. Phippard, A. Togias, S. R. Durham, M. H. Shamji

**Affiliations:** ^1^ Allergy and Clinical ImmunologyImperial College LondonLondonUK; ^2^ Immune Tolerance NetworkBethesdaMDUSA; ^3^ The National Institute of Allergy and Infectious DiseasesBethesdaMDUSA; ^4^ Immunomodulation and Tolerance groupImperial College LondonLondonUK

**Keywords:** cat allergy, nasal allergen challenge, tryptase, basophil, cytokine

## Abstract

**Background:**

Cat allergen is widely distributed in homes and schools; allergic sensitization is common.

**Objective:**

To develop a model of cat allergen nasal challenge to establish dose–response and time–course characteristics and investigate local and systemic biomarkers of allergic inflammation.

**Methods:**

Nineteen cat‐allergic individuals underwent titrated nasal challenge, range 0.243 to 14.6 μg/mL Fel d1, and matched diluent‐only provocation. Clinical response to 8 h was assessed by symptom scores and peak nasal inspiratory flow (PNIF). Nasal fluid was collected using polyurethane sponges and analysed by ImmunoCAP and multiplex assays. Whole blood flow cytometry for basophil surface CD63, CD107a, and CD203c was carried out at baseline and 6 h post‐challenge.

**Results:**

A dose–response to allergen was seen in symptom scores and PNIF, maximal at 10 000 BU/mL (4.87 μg/mL Fel d1), *P* < 0.0001 vs. diluent. Nasal fluid tryptase was elevated at 5 min after challenge (*P* < 0.05 vs. diluent); eotaxin, IL‐4, ‐5, ‐9, and ‐13 were increased at 8 h (*P* < 0.05 to *P* < 0.0001 vs. diluent); TSLP was undetectable; IL‐10, IL‐17A, and IL‐33 were unchanged compared to diluent challenge. Nasal fluid IL‐5 and IL‐13 correlated inversely with PNIF after challenge (IL‐5, *r* = −0.79, *P* < 0.0001; IL‐13, *r* = −0.60, *P* = 0.006). Surface expression of CD63 and CD107a was greater at 6 h than at baseline, both in the presence (both *P* < 0.05) and absence (CD63, *P* < 0.01; CD107a, *P* < 0.05) of *in vitro* allergen stimulation; no changes were seen on diluent challenge day.

**Conclusions:**

Cat allergen nasal challenge produces local and systemic Th2‐driven inflammatory responses and has potential as a surrogate outcome measure in clinical trials.

## Introduction

Exposure to cat allergen in societies such as the United Kingdom and USA where cats are common pets is virtually universal. Homes without cats have significant levels of cat allergen 1, as do day care centres 2, and schools 3. Such exposures are sufficient to impair asthma control 4. Sensitization rates are high: an estimated 17% of the US population is cat‐sensitized 5, increasing to 44% of inner city, moderate–severe asthmatic children 6. Accordingly, cat sensitization is estimated to account for 29% of asthma cases in the United States 5. Furthermore, avoidance measures, such as the use of HEPA filters in the home, have proved ineffective 7, whilst the very high levels of allergen found in some cat‐owning homes appear to have the potential to induce high‐dose tolerance 8.

Currently, the only potentially disease‐modifying treatment for cat allergy is allergen immunotherapy. Whilst use of this treatment is not uncommon, clinical studies of its efficacy and mechanisms are hampered by the difficulty of assessing symptoms in relation to allergen exposure. One solution has been the use of an environmental exposure chamber 9, recently demonstrating the efficacy of a cat allergen peptide immunotherapy. Unfortunately, such facilities are expensive and available in few centres.

Data from clinical studies concerning the systemic effects of nasal mucosal allergen exposure are limited. However, evidence from murine experiments and from human bronchial challenges suggests airway allergen exposure may cause mobilization of bone marrow inflammatory cell precursors, with trafficking back to sites of mucosal inflammation 10, 11, 12. Such systemic immune effects may account for the reciprocal inflammation that can be induced by allergen challenge at either the lower 13 or upper airway 14, and explain the benefit of intranasal corticosteroids on asthma control 15. Of note, mature, peripheral blood basophils have previously been shown to upregulate expression of the beta‐subunit of the high‐affinity IgE receptor after *in vivo* nasal allergen challenge in grass and ragweed allergic individuals 16.

We have previously developed a model of grass pollen nasal allergen challenge which provides reproducible clinical outcomes as well as measurement of inflammatory mediators in nasal fluid 17. Here, we attempt to use this model to study the effects of cat allergen nasal challenge, looking to establish dose–response clinical characteristics and identify local nasal biomarkers of the inflammatory response. Whilst the latter have been studied extensively in the context of seasonal allergens and house dust mite 18, there are limited data in the literature concerning the effects of cat allergen exposure. Importantly, we provide the first accurate time–course data out to 8 h after allergen challenge whilst controlling for any effects of diurnal variation on the parameters measured. Additionally, we investigated whether peripheral blood basophil activation, assessed by flow cytometry, could provide evidence of the systemic effect of nasal mucosal allergen exposure.

## Methods

### Participants

A total of 19 volunteers were recruited from amongst staff and students at The Royal Brompton Hospital and Imperial College, and from a database of previous study volunteers. Inclusion criteria were as follows: a history of at least 2 years of cat‐induced rhinoconjunctivitis (with/without mild asthma), a skin prick test to cat allergen ≥5 mm (Soluprick, ALK‐Abello, Hørsholm, Denmark), and a serum ImmunoCAP test to cat allergen of ≥0.70 KU/L. Exclusion criteria were as follows: a pre‐bronchodilator FEV_1_ < 70% predicted; uncontrolled or partly controlled asthma, or asthma requiring step 3 or above treatment according to Global Initiative for Asthma (GINA) guidelines 19; previous cat allergen immunotherapy; symptomatic seasonal allergic rhinitis during the study visits; and exposure to cat allergen at home or on more than an occasional basis elsewhere. The study was conducted outside of the UK tree and grass pollen seasons. Participants had not used nasal or inhaled corticosteroids or other anti‐allergy medications for at least 2 weeks prior to their visits for nasal challenge. The study was approved by the National Research Ethics Service, Surrey, and by the Research Office of The Royal Brompton and Harefield NHS Foundation Trust. Written informed consent was obtained before any study procedures were carried out.

### Study design

The study consisted of three visits. First, a screening visit where allergy history, medical history, spirometry, and skin prick testing to cat allergen were performed and blood taken for ImmunoCAP to major cat allergen Fel d1. Participants meeting the inclusion/exclusion criteria were then invited to return for two nasal challenge visits, at least 3 weeks apart. The first challenge involved administration of diluent only, the second increasing doses of cat allergen (Alutard SQ cat hair allergen extract, ALK‐Abello). On the day of each challenge, participants arrived at the Respiratory Biomedical Research Unit at The Royal Brompton Hospital between 8 and 9 a.m. After allowing 15 min for acclimatization, volunteers recorded their current nasal symptoms according to a verified scoring system: total nasal symptom score (TNSS) 20, a 12‐point scale with 4 categories: sneezing, nose running, nose blockage, and itching, each rated from 0 to 3. Additionally, the best of 3 peak nasal inspiratory flow (PNIF) measures using a nasal peak flow metre (Clement Clarke International, Harlow, UK) was recorded. Immediately afterwards, absorptive polyurethane sponges were placed into both nostrils to collect nasal mucosal fluid (see below). Following this, volunteers underwent a nasal lavage (Sinusrinse, NeilMed, Santa Rosa, California, USA). The above procedures were repeated 30 min after lavage. A further 10 min later, participants began the challenge (see below). TNSS, PNIF, and nasal fluid were recorded/collected at 5 min after the final challenge dose, then again at 15, 30, and 60 min, and at hourly intervals to 8 h. Participants remained within the unit the whole day. Additionally, 20 mL of peripheral blood was collected in lithium heparin tubes at least 15 min prior to challenge, then 6 h after the final challenge dose for the purpose of the peripheral basophil activation studies.

### Nasal allergen challenge

For the allergen challenge visit, cat allergen (Alutard SQ cat hair allergen extract, ALK‐Abello) was reconstituted at 100 000 SQ‐U/mL (equivalent to 30 000 BU/mL or 14.6 μg/mL of Fel d1) in albumin‐based diluent (ALK‐Abello). Dilutions in normal saline were then made at the following concentrations: 10 000 BU/mL (4.87 μg/mL), 5000 BU/mL (2.43 μg/mL), 1500 BU/mL (0.73 μg/mL), and 500 BU/mL (0.243 μg/mL). For the diluent challenge, albumin‐based diluent, in the absence of allergen, was diluted in normal saline in the same ratios as each concentration above. Two hundred and thirty microlitres of each concentration was added to disposable Bi‐dose nasal applicator devices (Aptar Pharma, Crystal Lake, IL, USA), manufactured to provide two 100 μL sprays. Each participant received one 100 μL spray of allergen to each nostril, applied by an examiner. The challenge began at 500 BU/mL, with increasing concentrations administered at 10‐min intervals, with response (TNSS and PNIF) recorded immediately before the next dose, continuing to 10 000 BU/mL (or, if the participant had not yet reached a TNSS score of 8 and was happy to proceed, and at the examiner's discretion, to 30 000 BU/mL). Participants were asked not to sniff strongly or blow their nose in the first 5 min after each allergen application.

### Collection and processing of nasal fluid

At each time point, after recording of TNSS and PNIF, a 20 × 15 × 5 mm piece of sterile synthetic polyurethane sponge (RG 27 grau, Gummi‐Welz GmbH & Co., Neu‐Ulm, Germany) was inserted by an examiner into each of the volunteer's nostrils, under direct vision using a Thuddicum's nasal speculum and croc forceps (both Phoenix Surgical Instruments Ltd, Hertfordshire, UK). Sponges were left in place for 2 min before removal and then added to centrifuge tubes with indwelling 0.22 μm cellulose acetate filters (Costar Spin‐X, Corning, NY, USA). Tubes were kept briefly on ice before being centrifuged at 4500 rcf at 4°C for 10 min. The isolated fluid was stored at −80°C. After thawing, nasal fluid was analysed for cytokines and chemokines using a human cytokine/chemokine magnetic bead panel 96‐well plate assay (Milliplex Map Kit, Millipore, MA, USA) and a Luminex xMAP Magpix platform (Millipore), according to the manufacturer's instructions. Samples of 25 μL of nasal fluid were analysed in duplicate alongside the manufacturer's standards and controls. The mean of the duplicate results was calculated after spurious results were excluded. Results below the lower limit of assay detection were given a value of 0. Tryptase and eosinophil cationic protein (ECP) in nasal fluid were measured using an ImmunoCAP 100 machine (Phadia/Thermo Scientific) according to the manufacturer's instructions. Nasal fluid samples were diluted 1 in 5 in assay diluent (ImmunoCAP IgE/ECP/Tryptase Diluent, Thermo Scientific) and run alongside calibrators and curve controls. Values below the lower limit of detection were given a value of 0.

### Whole blood basophil flow cytometry

Participant samples were immunostained within 90 min of phlebotomy, then analysed by flow cytometry. For each sample, 100 μL of heparinized blood was stained with the following fluorescent‐labelled antibodies, in the dark, at the manufacturer's recommended concentrations: anti‐CD203c PerCP‐Cy5.5, anti‐CD107a Pacific Blue, anti‐CD63 FITC (all BioLegend, London, UK), anti‐CD294 PE, anti‐CD303 APC (both Miltenyi Biotec, Bergisch Gladbach, Germany), and anti‐CD3 PE‐Cy7 (BD Biosciences, San Jose, CA, USA), or isotype controls, for 40 min. Red blood cells were then lysed by incubation for 10 min with 2 mL of BD lysing solution (BD Biosciences) in the dark. Samples were then centrifuged at 200 g for 5 min to remove supernatant and were washed with 3 mL of PBS. Cells were fixed using 450 μL Cell Fix solution (BD Biosciences) before analysis on a BD FACSCanto II flow cytometer (BD Biosciences). Basophils were identified as CD3^−^CD303^−^CRTH2^+^ cells (Figure S1). Additionally, two further 100 μL aliquots of each blood sample were immunostained as above, then incubated with anti‐human IgE (100 ng/mL) and cat hair allergen extract (100 μg/mL, Alutard SQ, ALK‐Abello), respectively, for 15 min at 37°C, before red cell lysis, fixation, and acquisition on the flow cytometer. Basophils were identified as CD3^−^CD303^−^CD294^+^ cells. Activation status was determined according to expression of CD63, CD107a, and CD207c.

### Statistical analysis

A commercial software package (Graphpad Prism Version 5.04, La Jolla, CA, USA) was used. For clinical outcomes (TNSS and PNIF) and basophil flow cytometry, analyses were made by paired *t*‐tests with Bonferroni correction for multiple comparisons where appropriate. For nasal fluid biomarkers, given the non‐normal distribution of the data, nonparametric statistics were used, with analyses by Wilcoxon matched‐pairs test or Mann–Whitney *U*‐test for unpaired data. *P*‐values < 0.05 were considered statistically significant.

## Results

### Participant demographics

Table 1 provides the demographic characteristics of the 19 participants. All participants completed both diluent and allergen challenge study visits.

**Table 1:** Individual participant demographic data and clinical response to active challenge (*n* = 19)

Age	22	26	27	32	25	26	54	45	33	34	57	44	28	27	29	22	22	21	48
Gender	F	M	F	F	F	F	F	F	M	M	F	M	F	F	F	F	F	F	F
Cat SPT (mm)	11.5	5	13.5	7	5	7.5	8	9	12	9.5	8	11	10	11	11.5	10	6	5	11
Fel d sIgE (kUA/L)	24.80	2.19	11.30	0.70	0.87	3.11	0.71	6.01	1.73	1.10	3.33	12.00	1.73	26.90	9.52	> 100	2.36	2.87	38.90
Total IgE (kU/L)	60.4	86.3	288	105	468	208	1102	339	148	116	60.8	495	743	311	370	1494	1140	6.87	338
Max. dose (BU/mL × 1000)	10	30	10	30	30	30	10	30	30	10	10	10	10	10	10	30	10	10	10
Peak score	7	9	10	8	8	9	8	7	10	9	10	9	8	8	10	8	9	8	8
Peak score dose (BU/mL × 1000)	5	30	5	5	30	30	5	30	30	5	10	5	5	10	5	10	5	10	1.5

SPT, skin prick test to cat hair allergen extract, mean diameter; Fel d sIgE, specific IgE to cat (Felis domesticus) allergen extract; Peak score dose, dose at which maximum TNSS score was reached; BU/mL, biological units per millilitre.

### Clinical responses to nasal challenge

**Figure 1:**
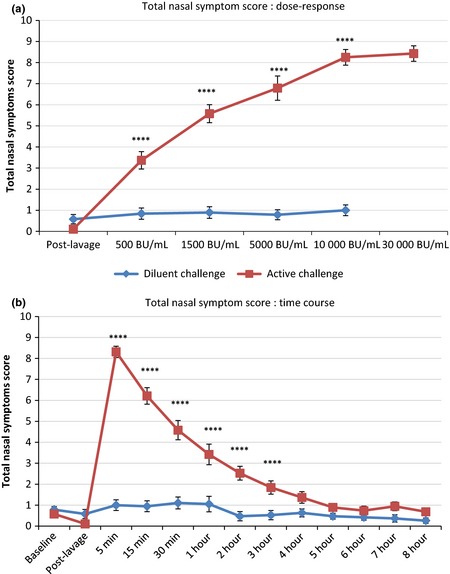
Total nasal symptom score (TNSS) response to active and diluent nasal challenge, mean ± standard error. (a) dose–response; (b) time–course response. ****, *P* < 0.0001, allergen vs. diluent, paired *t*‐test with correction for multiple comparisons.

The total nasal symptom score (TNSS) responses to diluent and allergen challenges are shown in Fig. 1. Seven of the 19 participants received a maximum allergen dose of 30 000 BU/mL, the rest 10 000 BU/mL (Fig. 1a and Table 1). One participant experienced a cough, without a change in peak expiratory flow, and did not go on to receive the 30 000 BU/mL dose. Otherwise, no participants were prevented from obtaining the top dose due to symptoms other than those of severe rhinoconjunctivitis. No response was seen to diluent challenge, whereas a significant response was seen to the first dose of allergen, 500 BU/mL, *P* < 0.0001 vs. diluent, with a progressive increase to 10 000 BU/mL; no further increase was seen at 30 000 BU/mL. Concerning the time–course of the response, scores were highest at 5 min after the final allergen dose, falling progressively until reaching similar levels to diluent challenge by 4 h (Fig. 1b).

**Figure 2:**
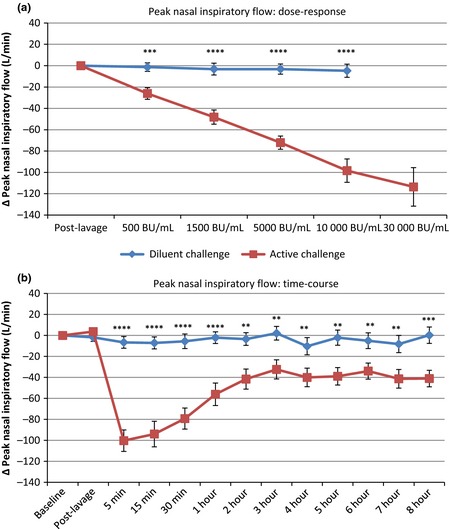
Peak nasal inspiratory flow (PNIF, change from baseline) response to active and diluent nasal challenge, mean ± standard error. (a) dose–response; (b) time–course response. **, ***, ****, *P* < 0.01, *P* < 0.001, *P* < 0.0001, respectively, allergen vs. diluent, paired *t*‐test with correction for multiple comparisons.

Peak nasal inspiratory flow (PNIF) values fell after allergen challenge, but not after diluent (Fig. 2). A significant fall was seen after the initial dose of 500 BU/mL, *P* < 0.001 vs. diluent, with progressive falls at each subsequent dose. PNIF values were lowest at 5 min after final allergen challenge, then gradually recovered up to 3 h, after which levels remained constant, and significantly reduced compared to diluent challenge, up to and including 8 h (*P* < 0.01 vs. diluent at 8 h, *P* < 0.0001 vs. baseline).

Peak expiratory flow rate (PEFR) did not change significantly during up‐dosing allergen challenge or over the following 8 h (Fig. S2). There were no correlations between participant cat‐allergen‐specific IgE levels or skin prick test size and any of: area under the curve for TNSS or PNIF, maximum TNSS, minimum PNIF, or threshold dose for a TNSS score of ≥7 (data not shown).

### Local nasal biomarker responses

**Figure 3:**
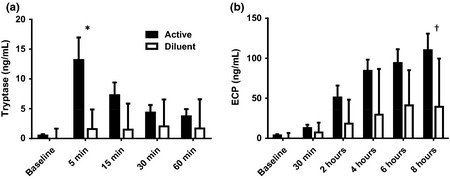
(a) Tryptase and (b) ECP levels in nasal fluid after cat allergen and diluent challenges, mean and standard error. *P < 0.05, active vs. diluent, 5 min; ^†^ *P* = 0.07, allergen vs. diluent, 8 h; both Mann–Whitney *U*‐test.

Nasal fluid tryptase levels peaked at 5 min after final allergen challenge, being significantly raised in comparison to diluent challenge and baseline (post‐lavage) (both *P* < 0.05, Fig. 3a). ECP levels continued to rise to 8 h post‐challenge (*P* < 0.0001 vs. baseline), showing a trend to an increase after allergen as compared to diluent (*P* = 0.07 vs. diluent, Fig. 3b).

**Figure 4:**
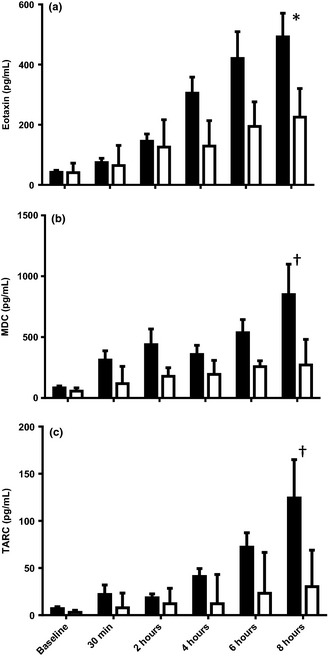
(a) Eotaxin, (b) MDC, (c) TARC levels in nasal fluid after cat allergen and diluent challenges, mean and standard error. *P < 0.05, ^†^ *P* < 0.09, allergen vs. diluent at 8 h, Mann–Whitney *U*‐test.

Eotaxin levels showed a progressive increase to 8 h post‐challenge (*P* < 0.05 and *P* < 0.0001 vs. diluent and baseline, respectively, Fig. 4a). MDC and TARC were also elevated after allergen compared to diluent at 8 h, narrowly missing statistical significance (both *P* = 0.09, Fig. 4b and c).

**Figure 5:**
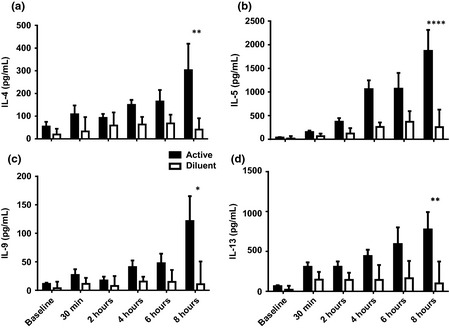
Th2 cytokines in nasal fluid following cat allergen and diluent nasal allergen challenge, mean and standard error. (a) IL‐4; (b) IL‐5; (c) IL‐9; (d) IL‐13. *, **, ****, *P* < 0.05, <0.01, <0.0001, respectively, 8 h, active vs. diluent, Mann–Whitney *U*‐test.

Levels of the Th2 cytokines IL‐4, ‐5, ‐9, and ‐13 progressively increased after nasal allergen challenge (Fig. 5). At 8 h, all were significantly elevated vs. diluent (IL‐9, *P* < 0.05; IL‐4 and IL‐13, *P* < 0.01; IL‐5, *P* < 0.0001) and baseline (IL‐4 and IL‐9, *P* = 0.01; IL‐13, *P* < 0.001; IL‐5, *P* < 0.0001).

Interleukin‐6 peaked at 8 h post‐allergen challenge and showed a trend towards an increase compared to diluent challenge (*P* = 0.07 vs. diluent at 8 h, Table S1). Levels of IFNγ, IL‐8, IL‐10, IL‐17A, IL‐33, and RANTES were not significantly different after allergen and diluent challenges (Table S1); TSLP was below the limit of detection in all samples; IL‐25 was within the detectable range in only one participant at a single time point (95 pg/mL, 30 min post‐allergen challenge).

### Clinical vs. biomarker responses

**Figure 6:**
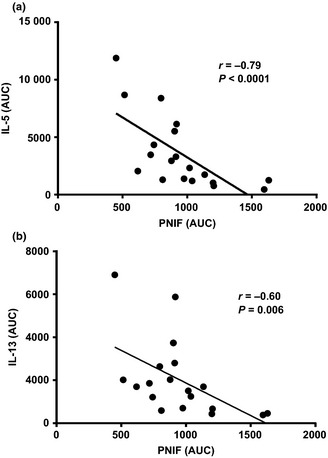
Peak nasal inspiratory flow (PNIF) area under curve (AUC) after allergen challenge vs. (a) IL‐5 AUC, and (b) IL‐13 AUC; correlations by Spearman's coefficient.

Levels of IL‐5 and IL‐13 in nasal fluid both showed an inverse correlation with peak nasal inspiratory flow after allergen challenge (IL‐5, *r* = −0.79, *P* < 0.0001; IL‐13, *r* = −0.60, *P* = 0.006, Fig. 6). Levels of IL‐5 and IL‐13 in nasal fluid correlated closely (*r* = 0.86, *P* < 0.0001, Fig. S3). A strong correlation was seen between IL‐9 and IL‐13 (*r* = 0.68, *P* = 0.001), and modest correlations between IL‐5 and both IL‐9 and eotaxin (*r* = 0.54, *P* = 0.02; *r* = 0.53, *P* = 0.02, respectively).

### Whole blood basophil flow cytometry

**Figure 7:**
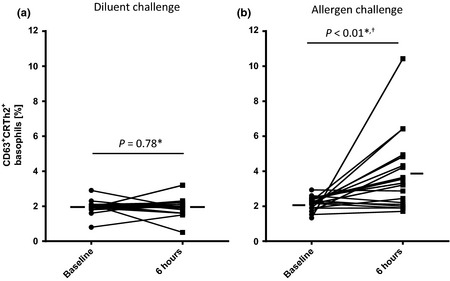
Proportions of CD63+ basophils before (baseline) and 6 h after diluent and allergen nasal challenges, in the absence of *in vitro* stimulation, assessed by flow cytometry. *baseline vs. 6 h, paired *t*‐test; ^†^ *P* < 0.001, 6 h post‐allergen vs. 6 h post‐diluent, paired *t*‐test.

No differences were seen in basophil CD63 surface expression before and 6 h after nasal challenge with diluent only (Fig. 7). Conversely, CD63 expression was increased 6 h after nasal allergen challenge compared to baseline both in the presence and absence of *in vitro* allergen stimulation (unstimulated basophils, *P* < 0.01; *in vitro* allergen‐stimulated basophils, *P* < 0.05, Fig. 7 and Table S2). In addition, CD63 expression in unstimulated basophils was greater at 6 h post‐allergen challenge than 6 h post‐diluent challenge (*P* < 0.001, Table S2). In common with CD63, expression of CD107a was greater at 6 h after nasal allergen challenge than pre‐challenge, both with and without *in vitro* allergen stimulation (both *P* < 0.05, Table S2). CD207a expression also increased after nasal allergen challenge, but only with *in vitro* allergen stimulation (*P* < 0.05, Table S2). No changes were seen between baseline and 6 h samples on diluent challenge day. Basophil CD63 expression did not correlate with clinical response to allergen challenge or local nasal biomarkers (data not shown).

## Discussion

This study demonstrates the dose–response and time–course characteristics of cat allergen nasal challenge combined with measures of local and systemic inflammatory responses. Nasal fluid tryptase is a marker of the allergic early phase response, with interleukins 4, 5, 9, 13 and the chemokine eotaxin markers of late phase inflammation. Their clinical relevance is illustrated by the inverse correlations seen between both IL‐5 and IL‐13, and peak nasal inspiratory flow after allergen challenge. There is no evidence of a significant contribution by epithelium‐derived mediators, with TSLP and IL‐25 levels generally below the detectable range, and no difference in IL‐33 between allergen and diluent challenges, nor does IL‐17A appear to exert a significant effect. Evidence of a systemic response to nasal allergen exposure is provided by the upregulation of blood basophil CD63 and CD107a, even in the absence of *in vitro* stimulation, not seen after diluent challenge.

Concerning the study design, the inclusion of a full diluent challenge day is justified by the laboratory results: nasal fluid mediators increased after allergen challenge, suggesting relevance to allergic inflammation; however, for several of these, no significant differences were seen between diluent and allergen challenge days. The relatively small but significant increases in markers of basophil activation post‐challenge might have been due to diurnal variation – that the same increases were not seen on the diluent day disproves this. The order of the challenges was not randomized to avoid residual inflammation at the second (allergen) challenge 21. As a result, diluent and active challenges were not blinded to the examiner or participants, meaning symptom scores were potentially prone to bias. However, the complete absence of symptoms on the control diluent day and the fact that clear immunological effects were only seen after active challenges support the validity of the clinical results, particularly the inverse correlations of nasal fluid IL‐5 and IL‐9 with peak nasal inspiratory flow; moreover, in our hands, we have found PNIF to be a reliable and reproducible objective physiological measure of nasal airway patency 17. Only non‐cat owners were recruited – chronic exposure might have abrogated the early phase responses, augmented non‐specific hypersensitivity, or, potentially, induced high‐dose tolerance. It remains to be seen if a cohort of cat‐owning/exposed individuals will respond in the same way. Whilst well‐controlled asthmatics were not excluded from the study, our final cohort did not include any patients currently taking inhaled corticosteroids; although no changes were seen in peak expiratory flow rate, we cannot absolutely assume this to be the case in cat‐allergic individuals with active asthma.

Several different approaches have been used to nvestigate cat allergy, most commonly a ‘cat room’ – usually a small‐sized room housing one or more cats. This has the advantage of simulating real‐life exposure, provoking both upper and lower airways with particles of multiple sizes 22, 23, but is inconvenient and gives highly variable levels of airborne allergen 22, 24. Nebulized allergen has been used to investigate bronchial reactivity, with reasonable correlation with cat room exposure 25. Nasal allergen challenge, in the form of drops, sprayed or soaked filter discs, has also been used 24, 26, 27, again correlating with cat room results 24. Environmental exposure chambers, supplying particulate allergen at tightly controlled levels, have also been used 9. Nasal allergen challenge has the advantage of allowing easy investigation of mucosal responses 17. Two studies have investigated the influence of cat allergen immunotherapy on local mediators, but no effects on IL‐4, ‐5, ‐10, IFNγ, or TGFβ were found, and baseline values were not reported 27, 28. Conversely, omalizumab reduced nasal fluid albumin 26 and PGD_2_ 29, although tryptase could not be measured reliably in the latter study 29. Our collection method – avoiding dilution of nasal fluid – and the improved sensitivity of commercially available assays have enabled a more detailed analysis of local mediators. The early rise in tryptase followed by gradual increases in Th2 cytokines and chemokines mirrors results of grass pollen nasal challenge 18. Finally, we also used an objective measure of nasal obstruction following challenge, peak nasal inspiratory flow. Acoustic rhinometry has been used in a previous cat challenge study, but results did not correlate with clinical symptoms 30.

The absence of a correlation between either serum cat allergen IgE or skin prick size and nasal challenge response is consistent with previous studies, both of the upper 24, 31 and lower respiratory tract 24, 25. Variations in non‐specific airways hyper‐responsiveness between individuals may be the missing component accounting for bronchial responses 32; whether an equivalent phenomenon is relevant in the upper airway is unclear.

Basophils have previously been investigated in the context of cat allergy. Paterniti et al. 31 found allergen‐induced basophil histamine release to be a predictor of response to nasal allergen challenge. Although not a precise equivalent, in this study, we did not find a correlation between basophil CD63, CD107, or CD207c expression and symptoms or peak nasal flow post‐challenge. Eckman et al. 29 found omalizumab decreased both response to nasal challenge and allergen‐induced basophil histamine release *in vitro*, suggestive of a pathological role for basophils *in vivo*.

Nasal allergen administration (grass or ragweed) over three days increased the expression of the beta‐subunit of the high‐affinity IgE receptor, FcεR1β, on mature basophils, as well as a trend to increased IL‐13 expression 16. This, alongside our results, highlights the systemic effect of nasal mucosal allergen exposure. Nasal allergen challenges have been shown to induce sinus 33 and lower respiratory tract inflammation in man 14, 34, as well as increased generation and trafficking of immune cells from the bone marrow in mice 10. Additionally, eosinophil transmigration capacity *in vitro* was increased at 2 h post‐nasal allergen challenge in man 35. These results provide a mechanistic basis for the beneficial effect of treatment of the upper airway on asthma inflammation and control 15, 36.

The mechanism by which nasal allergen provocation leads to activation of peripheral blood basophils is unclear. Possibilities include direct absorption of allergen from the nasal mucosa into the bloodstream (or swallowing of post‐nasal secretions and absorption via the gut mucosa, as suggested by a study using radio‐labelled *Parietaria judaica* allergen 37); migration of allergen‐activated antigen‐presenting cells to regional lymph nodes or into the circulation where they encounter effector cells; activation of effector cells in the mucosa followed by overspill into the circulation; or a systemic overspill of locally produced Th2 cytokines. Concerning the latter, elevated levels of IL‐5 and IL‐9 have been described in serum of allergic rhinitics compared to non‐atopic controls 38, 39; but, in contrast with nasal fluid, clear increases in serum Th2 cytokines in response to nasal allergen challenge have not, to our knowledge, been demonstrated. It seems improbable that the pg/mL levels detected in nasal fluid post‐challenge could have a significant effect once diluted in the circulation; moreover, basophil activation was increased at 6 h post‐challenge, prior to the peak in nasal fluid Th2 cytokine levels. Further investigations, including sensitive immunoassays of pre‐ and post‐challenge serum cytokine and grass allergen concentrations, and the ability of autologous serum taken pre‐ and post‐challenge to activate basophils *in vitro,* are needed to address these possibilities.

Our model shows a clear Th2‐dominant local response: tryptase marking the early phase response, interleukins 4, 5, 9, and 13 progressively increasing after allergen challenge, accompanied by eotaxin, plus strong trends to increases in MDC and TARC. Our results do not support a significant role for the Th1 cytokine IFNγ, the archetypal Th17 cytokine, IL‐17A, or epithelial‐derived cytokines in a pure allergen‐driven response. Evidence in the literature for a role for these latter cytokines in allergic rhinitis, in man at least, is also limited 18. Whether they may be of greater relevance following ‘natural’ exposure to cat allergen – exposure accompanied by other aeroallergens, pollutants, and microbes – requires further investigation. We have identified several potential local biomarkers which may provide mechanistic information on the action of anti‐allergy treatment, as well as a peripheral blood signal – basophil activation – which highlights the systemic immune consequence of mucosal allergen exposure.

Use of the clinical and laboratory techniques described here should be extended to interventional studies, including allergen immunotherapy, where they have potential to identify characteristics of responsive and unresponsive individuals, and highlight mechanisms of successful tolerance induction. Such studies may be conducted using up‐dosing challenges, similar to that described here, with the aim of identifying a shift in dose–response curve following treatment 27. Conversely, a single dose may be used, either tailored to each patient on the basis of a baseline titration challenge or a common fixed dose which would be expected to provoke significant symptoms in the majority of sensitized individuals with a clinical history. Evaluation of the late as well as early phase response in interventional studies would require a common fixed dose. In latter case, we would suggest either the 5000 BU/mL or the 10 000 BU/mL dose. This approach has the advantage of simplicity and speed. It also gives the cleanest time–course of response, with allergen administration at a single fixed point – particularly important if assessing early phase mediators. It remains to be determined whether these mediators are also predominant in cat‐allergen‐induced asthma, and whether, in patients with allergic asthma and rhinitis, effects on the upper respiratory tract might be used as surrogate for the lower airways.

In summary, we have investigated the dose–response and time–course of cat nasal allergen challenge in allergic, cat‐avoiding individuals and measured increases in nasal fluid tryptase during the early phase response, and Th2 cytokines and chemokines during late phase inflammation. Additionally, we provide real‐time evidence of a systemic immunological effect of nasal mucosal allergen exposure – the activation of peripheral blood basophils.

## Conflict of interest

The authors declare no conflict of interest.

## Supporting information


**Figure S1.** (a) Gating strategy for identification of basophils during whole blood flow cytometry. (b) identification of CD63‐expressing basophils, upper right quadrant, in the absence of *in vitro* allergen stimulation; (c) with *in vitro* allergen stimulation; d, stimulation *in vitro* with anti‐IgE monoclonal antibody.

**Figure S2.** Peak expiratory flow rate (PEFR) response to active and diluent nasal challenge, mean and standard error.

**Figure S3.** Correlations, by Spearman's coefficient, between Th2 cytokines/chemokines after allergen challenge.

**Table S1.** Cytokines/chemokines and ECP in nasal fluid after diluent and cat allergen challenges; mean (SE).

**Table S2.** Whole blood basophil flow cytometry at baseline and 6 h after diluent and allergen nasal challenges.
