# Supplementary figures and images for: Local and systemic effects of cat allergen nasal provocation
Source: Clin Exp Allergy. 2015 Feb 25;45(3):613–23. doi: 10.1111/cea.12434 (PMC4778413; doi:10.1111/cea.12434)

## Slide 1
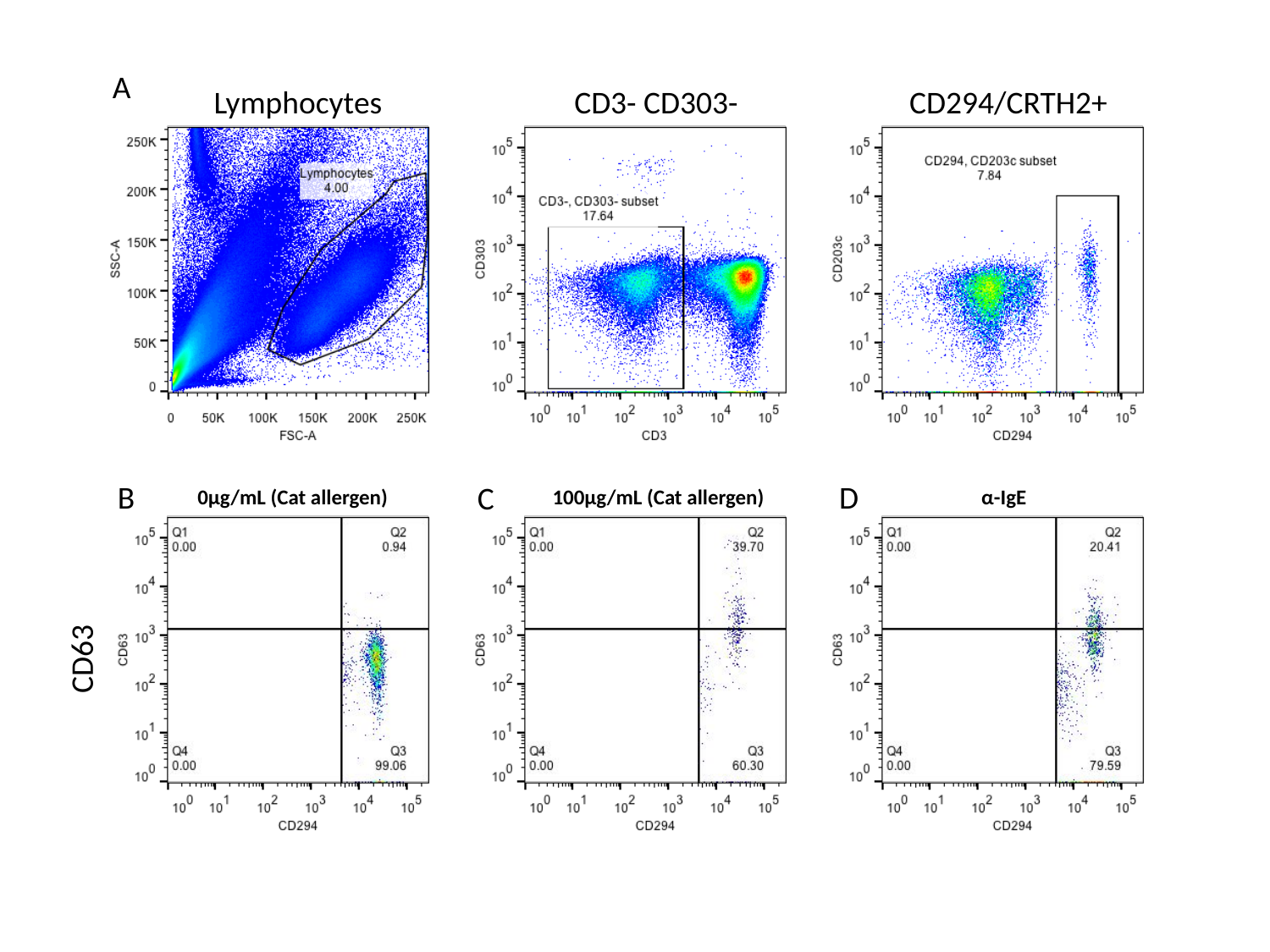

A
Lymphocytes
CD3- CD303-
CD294/CRTH2+
0μg/mL (Cat allergen)
100μg/mL (Cat allergen)
α-IgE
B
D
C
CD63

Supplement: Supplementary file 1 — Figure S1. (a) Gating strategy for identification of basophils during whole blood flow cytometry. (b) identification of CD63‐expressing basophils, upper right quadrant, in the absence of in vitro allergen stimulation; (c) with in vitro allergen stimulation; d, stimulation in vitro with anti‐IgE monoclonal antibody. [file CEA-45-613-s001.pptx]

## Slide 1
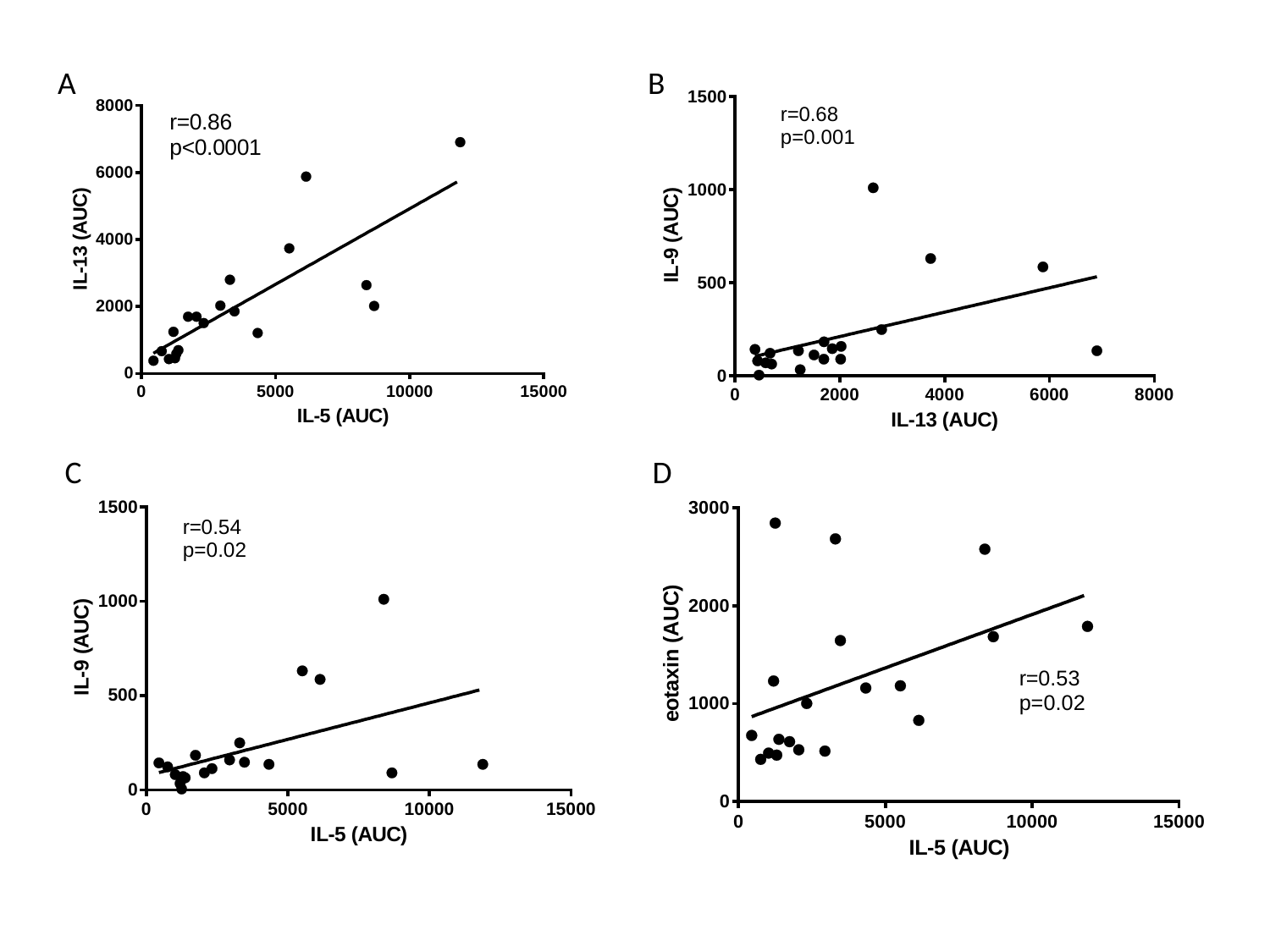

A
B
C
D

Supplement: Supplementary file 3 — Figure S3. Correlations, by Spearman's coefficient, between Th2 cytokines/chemokines after allergen challenge. [file CEA-45-613-s003.pptx]
