# Supplementary material for: Local and systemic effects of cat allergen nasal provocation
Source: Clin Exp Allergy. 2015 Feb 25;45(3):613–23. doi: 10.1111/cea.12434 (PMC4778413; doi:10.1111/cea.12434)
Supplement: Supplementary file 2 — Figure S2. Peak expiratory flow rate (PEFR) response to active and diluent nasal challenge, mean and standard error. [file CEA-45-613-s002.pptx]

## Slide 1
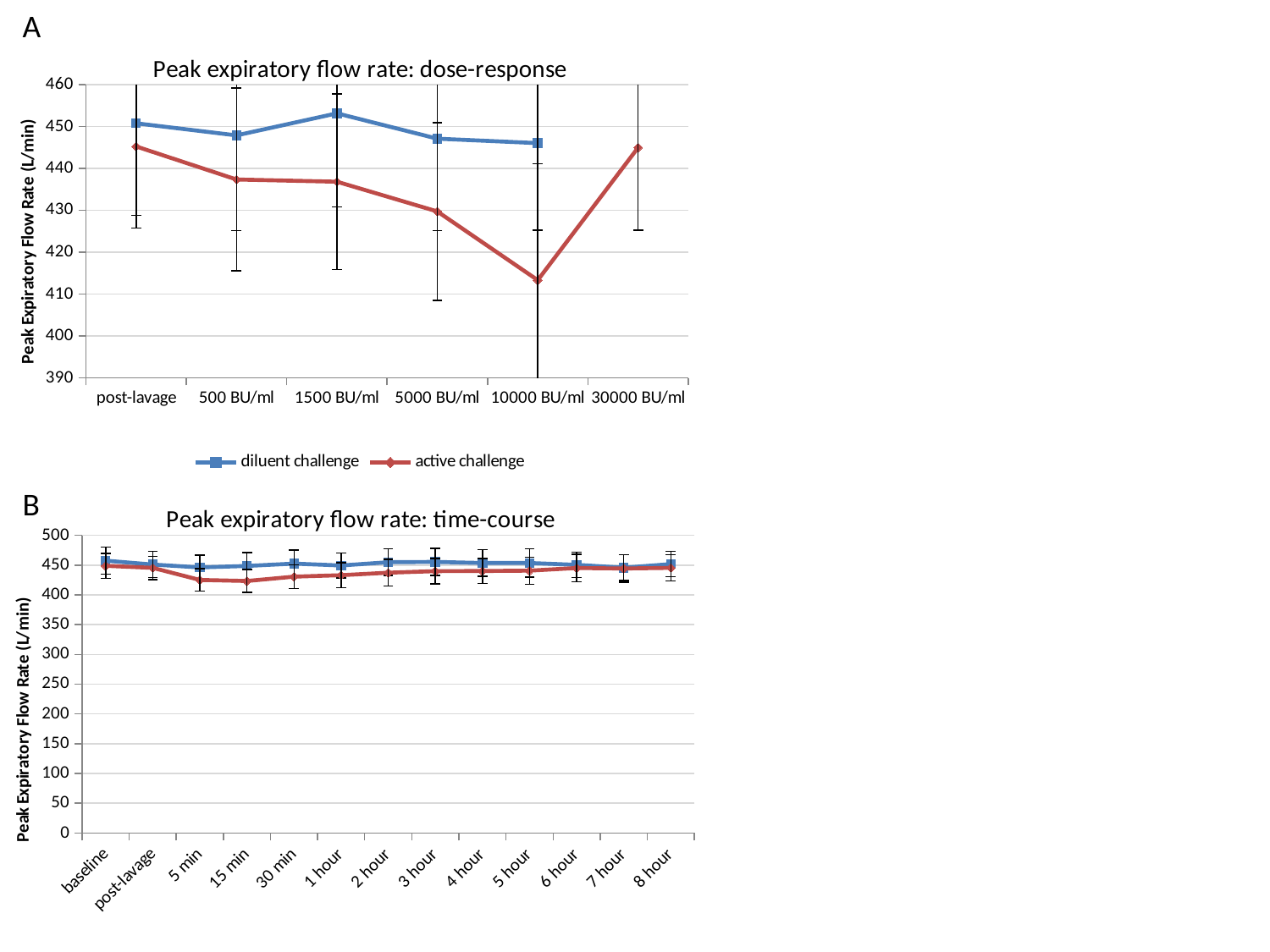

A
### Chart: Peak expiratory flow rate: dose-response
| Category | diluent challenge | active challenge |
|---|---|---|
| post-lavage | 450.7894736842105 | 445.2631578947368 |
| 500 BU/ml | 447.89473684210526 | 437.36842105263156 |
| 1500 BU/ml | 453.1578947368421 | 436.8421052631579 |
| 5000 BU/ml | 447.10526315789474 | 429.7368421052632 |
| 10000 BU/ml | 446.05263157894734 | 413.3333333333333 |
| 30000 BU/ml | None | 445.0 |
### Chart: Peak expiratory flow rate: time-course
| Category | diluent challenge | active challenge |
|---|---|---|
| baseline | 457.36842105263156 | 448.6842105263158 |
| post-lavage | 450.7894736842105 | 445.2631578947368 |
| 5 min | 446.05263157894734 | 425.0 |
| 15 min | 448.42105263157896 | 423.1578947368421 |
| 30 min | 452.36842105263156 | 430.5263157894737 |
| 1 hour | 449.2105263157895 | 432.89473684210526 |
| 2 hour | 454.7368421052632 | 437.10526315789474 |
| 3 hour | 455.2631578947368 | 439.7368421052632 |
| 4 hour | 453.42105263157896 | 440.0 |
| 5 hour | 453.42105263157896 | 440.5263157894737 |
| 6 hour | 450.2631578947368 | 445.0 |
| 7 hour | 445.5263157894737 | 444.2105263157895 |
| 8 hour | 451.57894736842104 | 445.2631578947368 |
