# Supplementary material for: Local and systemic effects of cat allergen nasal provocation
Source: Clin Exp Allergy. 2015 Feb 25;45(3):613–23. doi: 10.1111/cea.12434 (PMC4778413; doi:10.1111/cea.12434)
Supplement: Supplementary file 4 — Table S1. Cytokines/chemokines and ECP in nasal fluid after diluent and cat allergen challenges; mean (SE). [file CEA-45-613-s004.pptx]

## Slide 1
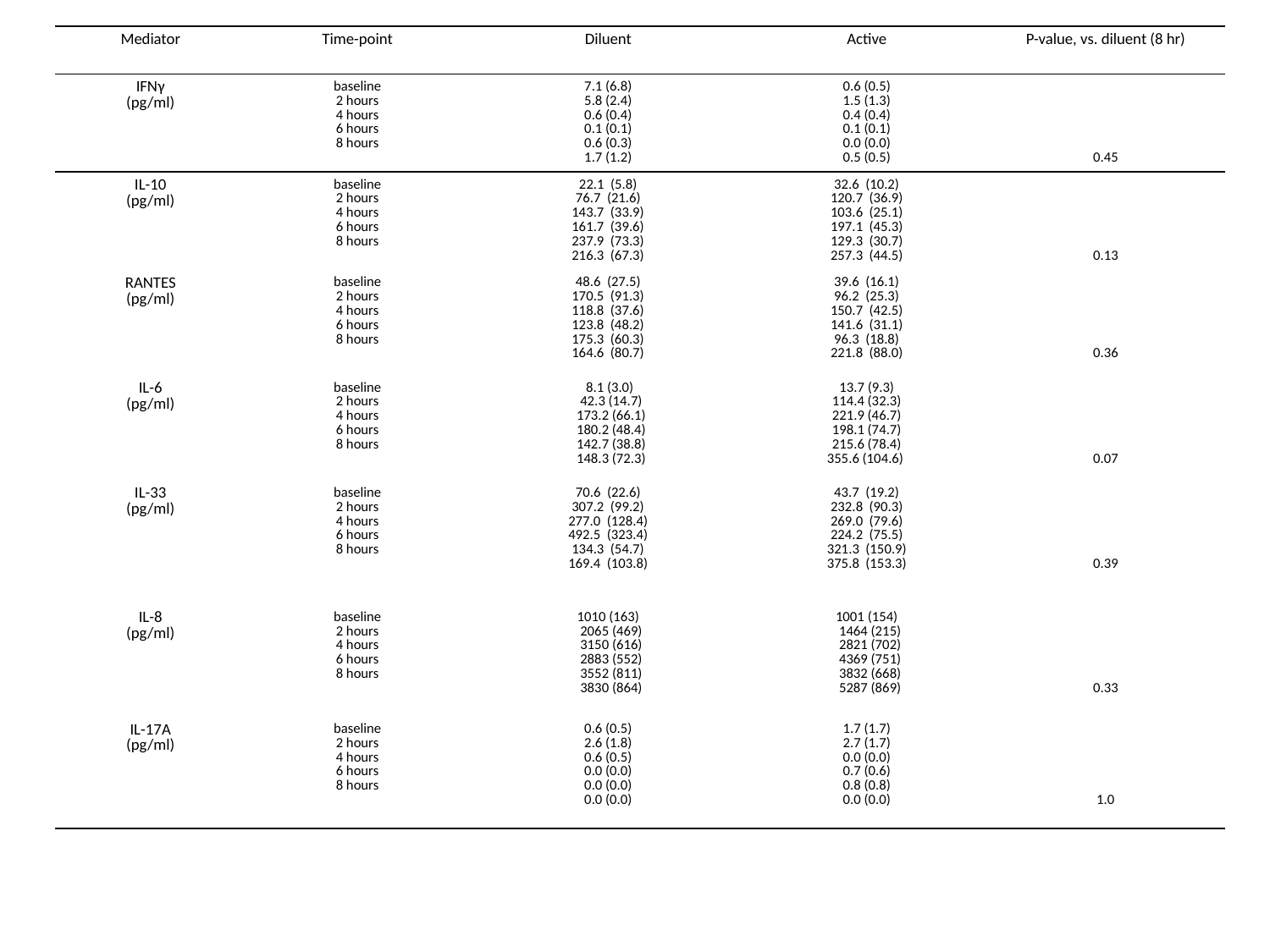

| Mediator | Time-point | Diluent | Active | P-value, vs. diluent (8 hr) |
| --- | --- | --- | --- | --- |
| IFNγ (pg/ml) | baseline 2 hours 4 hours 6 hours 8 hours | 7.1 (6.8) 5.8 (2.4) 0.6 (0.4) 0.1 (0.1) 0.6 (0.3) 1.7 (1.2) | 0.6 (0.5) 1.5 (1.3) 0.4 (0.4) 0.1 (0.1) 0.0 (0.0) 0.5 (0.5) | 0.45 |
| IL-10 (pg/ml) | baseline 2 hours 4 hours 6 hours 8 hours | 22.1 (5.8) 76.7 (21.6) 143.7 (33.9) 161.7 (39.6) 237.9 (73.3) 216.3 (67.3) | 32.6 (10.2) 120.7 (36.9) 103.6 (25.1) 197.1 (45.3) 129.3 (30.7) 257.3 (44.5) | 0.13 |
| RANTES (pg/ml) | baseline 2 hours 4 hours 6 hours 8 hours | 48.6 (27.5) 170.5 (91.3) 118.8 (37.6) 123.8 (48.2) 175.3 (60.3) 164.6 (80.7) | 39.6 (16.1) 96.2 (25.3) 150.7 (42.5) 141.6 (31.1) 96.3 (18.8) 221.8 (88.0) | 0.36 |
| IL-6 (pg/ml) | baseline 2 hours 4 hours 6 hours 8 hours | 8.1 (3.0) 42.3 (14.7) 173.2 (66.1) 180.2 (48.4) 142.7 (38.8) 148.3 (72.3) | 13.7 (9.3) 114.4 (32.3) 221.9 (46.7) 198.1 (74.7) 215.6 (78.4) 355.6 (104.6) | 0.07 |
| IL-33 (pg/ml) | baseline 2 hours 4 hours 6 hours 8 hours | 70.6 (22.6) 307.2 (99.2) 277.0 (128.4) 492.5 (323.4) 134.3 (54.7) 169.4 (103.8) | 43.7 (19.2) 232.8 (90.3) 269.0 (79.6) 224.2 (75.5) 321.3 (150.9) 375.8 (153.3) | 0.39 |
| IL-8 (pg/ml) | baseline 2 hours 4 hours 6 hours 8 hours | 1010 (163) 2065 (469) 3150 (616) 2883 (552) 3552 (811) 3830 (864) | 1001 (154) 1464 (215) 2821 (702) 4369 (751) 3832 (668) 5287 (869) | 0.33 |
| IL-17A (pg/ml) | baseline 2 hours 4 hours 6 hours 8 hours | 0.6 (0.5) 2.6 (1.8) 0.6 (0.5) 0.0 (0.0) 0.0 (0.0) 0.0 (0.0) | 1.7 (1.7) 2.7 (1.7) 0.0 (0.0) 0.7 (0.6) 0.8 (0.8) 0.0 (0.0) | 1.0 |
