# Supplementary material for: Local and systemic effects of cat allergen nasal provocation
Source: Clin Exp Allergy. 2015 Feb 25;45(3):613–23. doi: 10.1111/cea.12434 (PMC4778413; doi:10.1111/cea.12434)
Supplement: Supplementary file 5 — Table S2. Whole blood basophil flow cytometry at baseline and 6 h after diluent and allergen nasal challenges. [file CEA-45-613-s005.pptx]

## Slide 1
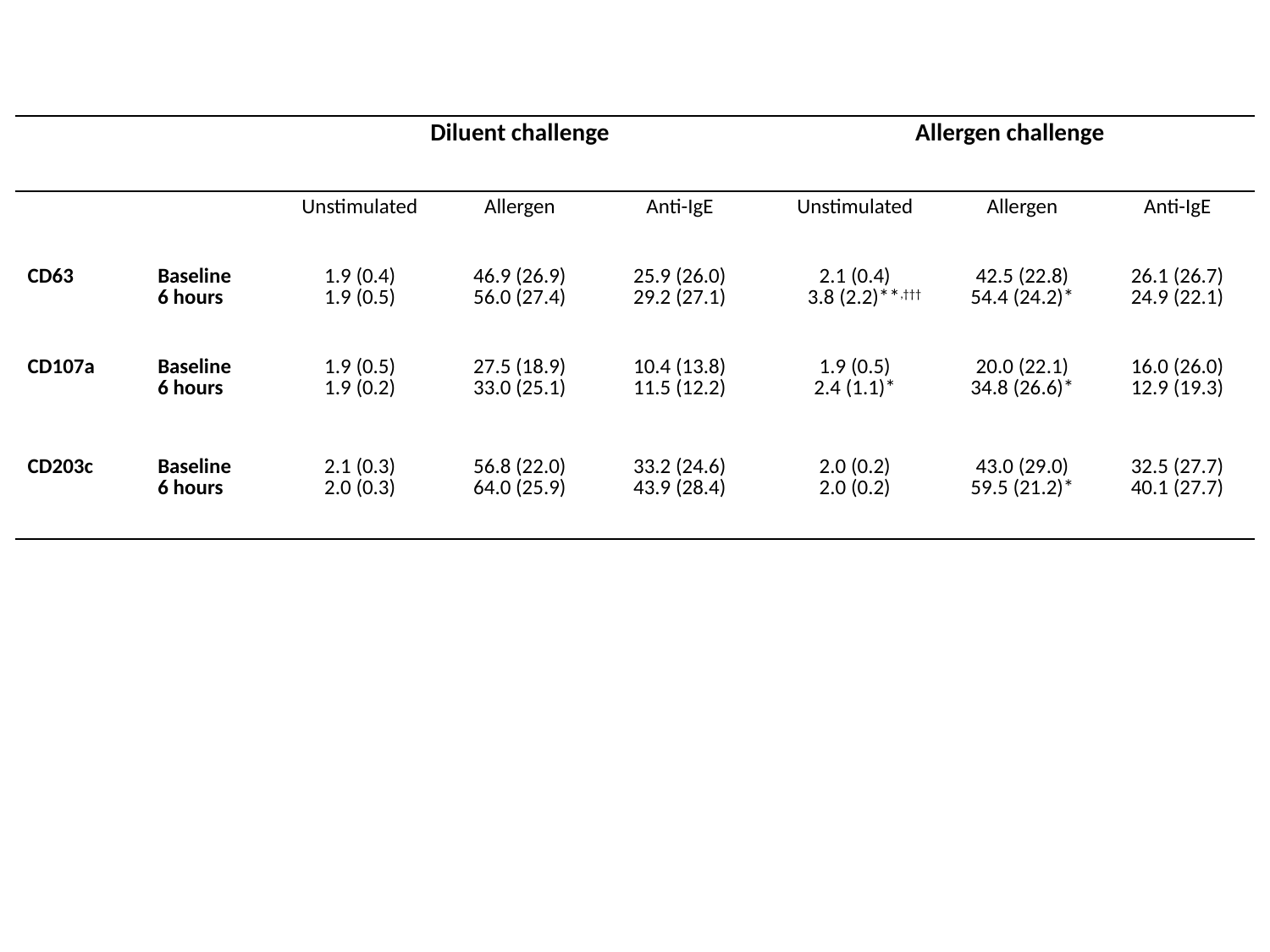

| | | Diluent challenge | | | Allergen challenge | | |
| --- | --- | --- | --- | --- | --- | --- | --- |
| | | Unstimulated | Allergen | Anti-IgE | Unstimulated | Allergen | Anti-IgE |
| CD63 | Baseline 6 hours | 1.9 (0.4) 1.9 (0.5) | 46.9 (26.9) 56.0 (27.4) | 25.9 (26.0) 29.2 (27.1) | 2.1 (0.4) 3.8 (2.2)\*\*,††† | 42.5 (22.8) 54.4 (24.2)\* | 26.1 (26.7) 24.9 (22.1) |
| CD107a | Baseline 6 hours | 1.9 (0.5) 1.9 (0.2) | 27.5 (18.9) 33.0 (25.1) | 10.4 (13.8) 11.5 (12.2) | 1.9 (0.5) 2.4 (1.1)\* | 20.0 (22.1) 34.8 (26.6)\* | 16.0 (26.0) 12.9 (19.3) |
| CD203c | Baseline 6 hours | 2.1 (0.3) 2.0 (0.3) | 56.8 (22.0) 64.0 (25.9) | 33.2 (24.6) 43.9 (28.4) | 2.0 (0.2) 2.0 (0.2) | 43.0 (29.0) 59.5 (21.2)\* | 32.5 (27.7) 40.1 (27.7) |
